# HUNT for better public health

**DOI:** 10.1177/14034948221102309

**Published:** 2022-09-14

**Authors:** Steinar Krokstad, Erik R. Sund, Kirsti Kvaløy, Vegar Rangul, Marit Næss

**Affiliations:** 1 HUNT Research Centre, Department of Public Health and Nursing, Faculty of Medicine and Health Sciences, NTNU, Levanger, Norway; 2 Levanger Hospital, Nord-Trøndelag Hospital Trust, Levanger, Norway; 3 Faculty of Nursing and Health Sciences, Nord University, Levanger, Norway

**Keywords:** Community surveys, public health, epidemiological methods, cohort studies, Norway

## Abstract

**Aims:**

The Trøndelag Health Study (HUNT) has collected population data through comprehensive decennial surveys over the last four decades and has so far collected data from 240,000 participants. The participants are identified with the unique Norwegian birth number, which enables them to be followed throughout different life stages, from survey to survey, and to endpoint measures in Norwegian national health registers without attrition bias.

**Methods:**

The study design of HUNT offers several advantages: it provides an overview of the public health development in the population over decades, the data can be used in health services research, clinical epidemiology, studies of causation, trajectories, and consequences of diseases, and to study gene × environment interactions.

**Results:**

HUNT data have shown major shifts in public health trends, such as decreasing mean blood pressure and resting heart rate among adults, increasing prevalence of obesity, geographical and socioeconomic inequalities in health, increasing mental health distress among adolescents and young adults with an opposite development among the elderly. Data from HUNT have been used in several major international research projects, where data harmonization with several other population cohorts internationally has been done. HUNT has placed great emphasis on safeguarding research ethics, privacy, and data security. The Norwegian authorities established national regulations for the surveys from the time General Data Protection Regulation was introduced in 2018.

**Conclusions:**

**Researchers can apply for HUNT data access from HUNT Research Centre provided they have obtained project approval from the Regional Committee for Medical and Health Research Ethics. Researchers not affiliated to a Norwegian research institution must collaborate with and apply through a Norwegian principal investigator. Information on the application and conditions for data access is available at www.ntnu.edu/hunt/data.**

## The HUNT Study

**Figure 1.**
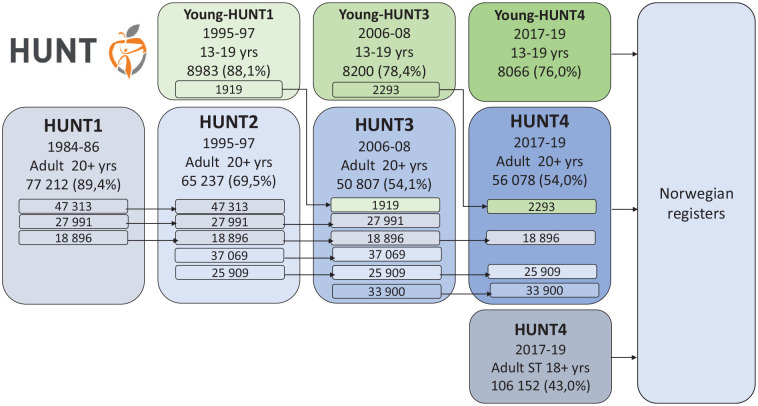
The HUNT surveys, time-periods, number of participants, number of participants attending two or more surveys, and attendance (%). Surveys conducted in the northern region, Nord-Trøndelag (NT): HUNT1 (1984–1986), HUNT2 (1995–1997), HUNT3 (2006–2008), HUNT4 (2017–2019), Young-HUNT1 (1995–1997), Young-HUNT3 (2006–2008), Young-HUNT4 (2017–2019). Young-HUNT2 was a follow-up of a subsample from Young-HUNT1 in 2000-2001 (not shown). Survey conducted in the southern region, Sør-Trøndelag (ST): HUNT4 (2017–2019). yrs: years

Along with the existence of SJPH (initiated in 1973), HUNT (The Trøndelag Health Study) has collected valuable population data through comprehensive surveys over four decades (Figure 1). The unique feature concerning HUNT is that *all residents* in the northern part of Trøndelag County are invited by mail to participate in the surveys for adults (20+ years) [1,2] and young people (13–19 years) [3]. The health examinations are decentralized and take place in field stations in all municipalities, middle schools and high schools in this region. The adult population (18+ years) of the southern part of the county was additionally invited in the last wave in a questionnaire survey. The participants are identified with the unique Norwegian birth number, which enables them to be followed throughout different life stages, from survey to survey, and to endpoint measures in Norwegian national health registers without attrition bias. Figure 1 shows that 18,896 adults participated in all four surveys. The surveys include questionnaires, interviews, clinical tests and collection of biological material (blood, urine, faeces, saliva). An overview of the data material is published by HUNT Databank online (www.hunt-db.medisin.ntnu.no/hunt-db/).

The study design of HUNT offers several advantages: it provides an overview of the public health development in the population over decades, as the data show social, regional and local variations in health [4,5]. The data can be used in health services research [6,7], clinical epidemiology, studies of causation, trajectories and consequences of diseases, and to study gene × environment interactions. Studies of molecular mechanisms of disease have also been conducted [8]. Repeated measurements from adolescence to adulthood or from young adulthood to later adulthood offers multiple opportunities for longitudinal studies. HUNT participants with linkage to family data facilitate larger intergenerational studies of inheritance and the impact of nature versus nurture on selected traits and health-related relationships of interest. Finally, genetic data are now available on adult participants from HUNT2, 3 and 4, and enable us to better understand the genetic basis of diseases in the population [9].

## Public health data

HUNT data have shown major shifts in public health trends, such as decreasing mean blood pressure and resting heart rate among adults [10,11], increasing prevalence of obesity [4,12,13], geographical and socioeconomic inequalities in obesity [4,13], increasing mental health distress among adolescents and young adults with an opposite development among the elderly [14], socioeconomic inequalities and trends in risk factors [15]. The potential for following and understanding trends in public health development is excellent with data from several decades. In the latest survey (HUNT4) we collected more objective data, for body composition (bioelectrical impedance analysis) and objective measures of physical activity and sedentary behaviour using accelerometers. These data and linking data to geographic information system data offer exciting opportunities in how our surroundings and environment affect lifestyle and thereby public health.

National, regional and local authorities have shown great interest in the data and the results provide a basis for public health policy, health-promoting and preventive efforts, and health services planning and strategies [5,16,17]. In addition to findings on a national level, the HUNT data have also been used in international studies. HUNT data have been used to calculate disease burden [18] as well as contribute to global initiatives concerning health trends [19]. Data from HUNT have been used in several major international research projects [18–20], where data harmonization with several other population cohorts internationally has been an issue [19,21,22]. Thus, the HUNT Study is central to Nordic public health research. The Nordic countries are unique in a global context because the countries have developed strong public welfare schemes. Data from HUNT can shed light on these schemes’ significance for public health; disease, illness, sickness, health-related behaviour, quality of life, social and geographical health inequalities, in addition to following public health trends in a Nordic population compared with trends in other populations [23] or regime types internationally [13].

## Ethics, privacy, data security

From the very beginning, HUNT has placed great emphasis on safeguarding research ethics, privacy [24] and data security by International Organization for Standardization certification of HUNT Databank/Biobank and HUNT Cloud. To ensure that HUNT and other valuable population-based health surveys can contribute to knowledge about the population’s health now and in the future, the Norwegian authorities established national regulations for the surveys from the time General Data Protection Regulation was introduced in 2018 (https://lovdata.no/dokument/LTI/forskrift/2018-04-27-645).

## How can public health data be accessed by researchers?

Researchers affiliated to a Norwegian research institution can apply for HUNT data access from HUNT Research Centre (www.ntnu.edu/hunt) provided they have obtained project approval from the Regional Committee for Medical and Health Research Ethics. Researchers not affiliated to a Norwegian research institution must collaborate with and apply through a Norwegian principal investigator. Information on the application and conditions for data access is available at www.ntnu.edu/hunt/data.
